# Graduated compression stockings as a prophylactic measure in venous thromboembolism and edema of lower limbs triggered by air travel: a systematic review of clinical trials

**DOI:** 10.1590/1677-5449.200164

**Published:** 2021-05-10

**Authors:** Lorenna Ferreira da Silva, Melissa Stephane Roberta Porto, Albert Bacelar de Sousa, Katia de Miranda Avena

**Affiliations:** 1 Centro Universitário UNIFTC, Salvador, BA, Brasil.

**Keywords:** pulmonary embolism, air travel, stockings, compression, venous thrombosis, disease prevention, patient safety

## Abstract

The increase in duration and frequency of flights has led to an increase in the prevalence of venous thromboembolism among airline passengers. This study assesses the efficacy of graduated compression stockings for prevention of venous thromboembolism triggered by flights lasting more than 3 hours. The design is a systematic review of clinical trials. The methodological quality of studies and the level of scientific evidence were evaluated using the Consolidated Standards of Reporting Trials and Grading of Recommendations Assessment, Development and Evaluation standards. A total of 34 articles were identified, but only eight met the eligibility criteria. The outcomes incidence of venous thromboembolism and edema were assessed in 2,022 and 1,311 passengers, respectively. The studies presented high quality evidence demonstrating prevention of edema and moderate quality evidence of reduced incidence of venous thromboembolism associated with wearing graduated compression stockings during flights.

## INTRODUCTION

Air travel is one of the most widely used forms of transport, carrying billions of people per year globally. Over the last decade, the number of journeys made by air rose constantly, in line with increasing numbers of flights offered; and more people are therefore traveling by air. Studies show that 3.8 billion people travel by air annually and that 300 million of them take long-duration flights,^1^^-^ 3 thereby increasing the risk of diseases associated with flights lasting more than 3 hours, the most important of which are deep venous thrombosis (DVT) and pulmonary thromboembolism (PTE).^4^

Both DVT and PTE are part of the spectrum of a single disease: venous thromboembolism (VTE). DVT primarily occurs in the lower limbs, caused by coagula that form in the deep vein system. When one of these coagula becomes detached, it travels to the right cardiac chambers and then to the pulmonary circulation, causing reduction or cessation of blood flow to pulmonary segments. This characterizes PTE, which can manifest with presentations including dyspnea, pleural pain, and hemoptysis. VTE can also cause chronic complications, such as pulmonary hypertension and postthrombotic syndrome.^5^^-^ 7

According to Virchow’s triad,^5^ three factors can contribute to development of VTE, in isolation or in conjunction: blood flow stasis, hypercoagulability, and endothelial injury.^8^^,^ 9 The global rate of flight-related PTE is still relatively low, with an incidence of 3.2 cases/1,000 people/year. This incidence is around 3.2 times greater than in the healthy population that does not travel by air.^10^ Moreover, the risk of PTE development can also double on flights lasting more than 8 hours and increases 26% for every extra 2 hours of duration.^11^ The risk of severe PTE is illustrated by an incidence of approximately 0.4 cases per million passengers.^12^

Development of VTE during flights has been primarily associated with temporary lower limb immobility caused by remaining sitting for extended periods, increasing venous stasis and reducing blood flow to the deep and superficial veins of the lower limbs.^13^ Other factors include diseases or conditions that predispose to formation of DVT or PTE, such as prior history of VTE and thrombophilias, among others.^5^

Graduated compression stockings (GCS) constitute a non-pharmacological method for reducing the risk of VTE in cases in which there is prolonged immobility, because they reduce venous stasis by increasing venous blood flow in the lower limbs.^13^ In view of the increasing frequency of air travel and the consequent increased risk of VTE development, studies have been conducted to attempt to identify safe and effective prophylactic measures. In this context, one possible strategy for prevention of these diseases is wearing GCS during flights.

Edema is another relevant occurrence on long-duration flights, affecting both patients with venous diseases and healthy people. Studies have shown that edema can be observed in almost all people who fly for more than 7 hours, occurring in around 97% of passengers and being more evident in patients with chronic venous disease.^14^

In view of the above, the objective of this article is to identify the existing scientific evidence on wearing GCS during flights as a prophylactic measure against VTE and formation of edema in the lower limbs.

## METHODS

A systematic review of the literature was conducted according to the following logical sequence: 1) definition of the research question using the PICOT strategy (Patient or Problem, Intervention, Control or Comparison, Outcomes); 2) identification of databases for study selection; 3) definition of inclusion and exclusion criteria; and 4) evaluation of the methodological quality of the articles identified.

Since this is a study of secondary data available in the published literature, with no primary experimentation on human beings, submission to a Research Ethics Committee was waived.

### Search strategy

The research question defined to guide the search strategy was: “Is wearing GCS during flights an effective prophylactic measure to protect against development of VTE and edema of the lower limbs?” Scientific articles were identified by searching the PubMed and Biblioteca Virtual de Saúde platforms. Using standard health descriptors (DECs and MeSH), the following keywords (and their equivalents in Portuguese) were chosen: *“Pulmonary embolism”, “Air travel”, “Stockings, Compression”*. These keywords were combined as follows for PubMed: “Pulmonary embolism” AND “Air travel” AND “Stockings, Compression”. This search strategy was then adapted to suit the Biblioteca Virtual de Saúde. The last update was on April 30, 2020.

As an intentional strategy, more comprehensive terms were used initially, with the aim of identifying a large number of studies and thereby minimize the chances of missing an important article in the search. A manual search was also performed of the lists of references in the original list of studies identified, with the objective of identifying relevant studies that met the eligibility criteria, but which for some reason had not been returned in the results of the search strategy chosen.

### Eligibility criteria

No restrictions were imposed in terms of date or language of publication. Since the study is a systematic review of randomized clinical trials, books, book chapters, editorials, narrative and systematic reviews, and other types of texts that do not go through the rigorous peer review process to which scientific articles are subjected were all excluded. Duplicated texts indexed in more than one database were also excluded.

### Data collection, classification, and evaluation of study quality

The incidence of asymptomatic/symptomatic VTE and presence of edema in the lower limbs when wearing or not wearing GCS on flights were defined as the variables of interest to be reviewed in the study. The bibliographic data were collected from February 1 to April 30 of 2020. After identification, studies were subjected to critical assessment. This step consisted of reading and analysis of the titles and abstracts, conducted by two independent reviewers. In this step, studies were excluded that did not fit the objectives of the systematic review. After this initial screening, the next step consisted of reading the full texts of studies, leading to exclusion of some texts that were not judged to be reliable (for issues of methodology, randomization, conflicts of interest, and other factors). Finally, the studies were organized in a table in order to present their principal details, facilitating a descriptive and critical analysis of the results reported by their authors.

Methodological quality of studies was evaluated according to the Consolidated Standards of Reporting Trials (CONSORT),^15^ a checklist comprising 25 items to provide an assessment of the methods, analysis, and validity of the results presented in the randomized clinical trials included in the review. The Grading of Recommendations Assessment, Development and Evaluation (GRADE) system was used to classify the quality of scientific evidence and strength of recommendations.^16^ The GRADE system assesses the quality of evidence for each outcome analyzed, classifying it at one of four levels: high, moderate, low, or very low. The evidence level is determined by considering the following factors: study design; methodological limitations (risk of bias); inconsistencies; indirect evidence; imprecision; publication bias; effect size; dose-response gradient; and residual confounding factors.

## RESULTS

A total of 34 articles were identified, 14 of which were excluded after reading the titles. One of the 20 articles remaining was excluded for not meeting the eligibility criteria after reading the abstracts. The remaining 19 articles were selected for reading of the full text. Another 11 texts were excluded after reading the full text, also for not meeting eligibility criteria. The final sample therefore comprised eight articles.

**Figure 1:**
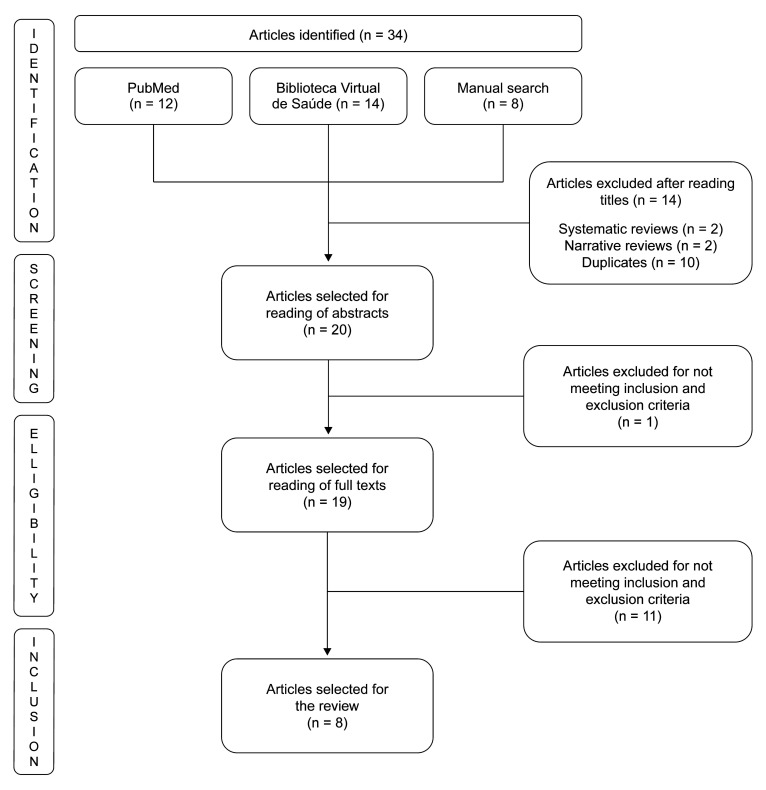
Flow diagram illustrating article selection.

Figure 1 shows the flow diagram illustrating the number of articles found and selected after application of inclusion and exclusion criteria. After the articles had been selected, the basic characteristics of the eight eligible studies were listed in Tables 1 and 2, facilitating a descriptive and critical analysis of the results presented by their authors. Table 3 contains the results of assessment of the methodological quality of the randomized clinical trials using the CONSORT instrument, considering their methods and analysis and the validity of the results presented. The quality of evidence for the outcomes assessed by the studies included in the systematic review is presented in Table 4. After analysis using the GRADE system, it was observed that there is a high/moderate degree of confidence in the effects estimated, but future randomized clinical trials may change confidence in the estimate of the effect.

**Table 1:** Summary of the main descriptive characteristics of the studies reviewed.

Author (year)	Study location	Details of sample	Method
Scurr et al. (2001)^17^	London, UK	231 passengers (89 men and 142 women), aged over 50 years, with no history of thromboembolic problems who intended to travel in economy class for at least 8 h in 6 weeks.	Random allocation of passengers into two groups: one group wore class I below-the-knee GCS (20-30 mmHg according to the German Hohenstein compression standard) and the other group did not.
		31 were excluded because of health problems, changes to travel plans, or inability to fulfill commitments.	Flow duration was measured by color or pulsed Doppler ultrasound. Duplex USG was used to assess deep veins before and after the journey by a technician blind to which group the volunteer had been randomized into. Presence of current venous disease or prior thrombosis was assessed by B mode imaging and color flow mapping and veins were assessed with B mode imaging. Blood samples were analyzed for two specific common genetic mutations that predispose to venous thromboembolism, factor V Leiden (FVL) and prothrombin G20210A (PGM), using the PCR technique. A sensitive D-dimer assay was used to screen for recent development of thrombosis.
			In the first 30 volunteers, USG examinations were performed 2 weeks before the journey and again within 2 days of the start of the first flight to provide a control for the interval during which occurrence of spontaneous DVT could be assessed in this population.
Belcaro et al. (2002)^18^	Italy, ITA	657 passengers of both sexes with moderate DVT risk.	PART I (7-8 h flights): 372 participants randomized into two groups: control group with no prophylaxis and treatment group wearing specially designed GCS (Flight Socks, Scholl producing 14-17 mmHg of pressure at the ankle). High risk criteria: previous episodes of DVT or superficial venous thrombosis, coagulation disorders, severe obesity, limited mobility caused by bone or joint problems, oncological disease during the preceding 2 years, cardiovascular disease, or large varicose veins. Individuals with height exceeding 190 cm and/or weight exceeding 90 kg were excluded. USG scanning protocol (before and after the flight): Sonosite 7.5 to 13 MHz scanners with high resolution linear probes were used to examine the venous system while compressing the major veins (popliteal, femoral). Suggestions for passengers were given to both groups (perform mild exercises, such as walking; drink water and avoid salty foods; avoid excessive baggage that restricts legroom). Edema assessment was based on the edema tests, ankle circumference, and discomfort score.
		PART I: GCS group: 101 men and 78 women, mean age of 49.0+7.0 years. Control group: 98 men and 81 women, mean age of 48.4+7.3 years.	PART II (11-12 h flights): 285 participants randomized into two groups: a control group with no prophylaxis and a treatment group wearing below-the-knee GCS (Flight Socks, Scholl, UK, producing 14-17 mmHg of pressure at the ankle). The same procedures and criteria were adopted as had been used in part I.
		PART II: GCS group: 89 men and 53 women, mean age of 48.0+8.0 years. Control group: 87 men and 56 women, mean age of 47.0+8.0 years.	
Cesarone et al. (2003)^19^	Italy, ITA	284 low-medium risk passengers of both sexes in parts I and II.	PART I (7-8 h flights): 144 participants randomized into two groups: the control group had no prophylaxis and the treatment group wore GCS (Kendall, Travel Socks, Tyco Healthcare, Mansfield, United States) producing 20-30 mmHg of pressure at the ankle, with pressure reducing at the mid-calf. Suggestions for passengers were given to both groups (perform mild exercises, such as walking; drink water and avoid salty foods; avoid excessive baggage that restricts legroom). Ultrasound scanning protocol (before and after the flight): Sonosite 7.5 to 13 MHz scanners with high resolution linear probes (Sonosite, Bothell, United States) were used to examine the venous system while compressing the major veins (popliteal, femoral, tibial). Edema was assessed using a score proposed by Valentino and Irvine, based on a combination of data on ankle circumference (cm) and volume (mL) combined with subjective assessments of edema and discomfort.
		PART I: GCS group: mean age of 46+8.0 years, 37 men and 35 women. Control group: mean age of 47.0+5.0 years, 38 men and 34 women.	PART II (11-12 h flights): 132 participants randomized into two groups: the control group had no prophylaxis and the treatment group wore Kendall below-the-knee GCS. 30 participants with edema-related microangiopathy (10 with diabetes, 10 with chronic venous insufficiency and venous hypertension, and 10 on antihypertensive treatment with ACE inhibitors for hypertension) were included to evaluate the effects of GCS during the flights. The same procedures and criteria were adopted as had been used in part I.
		PART II: GCS group: mean age of 47.0+5.0 years, 34 men and 30 women. Control group: mean age of 46.9+4.0 years, 34 men and 32 women.	
Cesarone et al. (2003)^20^	London, UK	PART I: 266 individuals at low-medium risk of DVT, with 55 excluded for non-medical problems related to their journeys.	PART I (7-8 h flights): 211 participants randomized into two groups: the control group had no prophylaxis and the treatment group wore below-the-knee GCS (Traveno, Sigvaris, Brazil), producing 12-18 mmHg of pressure at the ankle. Criteria for risk of DVT: previous episodes of DVT or superficial thrombosis, coagulation disorders, severe obesity, limited mobility caused by bone or joint problems, oncological disease during the preceding 2 years, cardiovascular disease, or large varicose veins. Individuals with height exceeding 190 cm and/or weight exceeding 90 kg were excluded. Ultrasound digitalization protocol (before and after the flights): Sonosite 7.5 to 13 MHz scanners with high resolution linear probes were used to examine the venous system while compressing the major veins (popliteal, femoral). Suggestions for passengers were given to both groups (perform mild exercises, such as walking; drink water and avoid salty foods; avoid excessive baggage that restricts legroom). Edema was assessed based on the edema test, ankle circumference, edema and discomfort score. The combined edema score was developed based on assessment of parametric data, such as the edema test, change in ankle circumference (cm), volume measurements (in mL or percentage volume), combined with subjective assessment of edema and discomfort (from 0 to 10) defined directly by participants before and after the flights.
		PART II: 200 low-medium risk individuals, with 35 excluded for non-medical reasons.	PART II (11-12 h flights): the remaining 165 participants were randomized into two groups: the control group had no prophylaxis and the treatment group wore below-the-knee Traveno GCS. The same procedures and criteria were adopted as had been used in part I.
Belcaro et al. (2003)^14^	Italy, ITA	300 individuals at high risk of DVT were contacted and pre-included; 76 were later excluded on the basis of a variety of considerations and the remainder were randomized into two groups (GCS, n = 110, vs. controls, n = 114) to assess prophylaxis with below-the-knee GCS.	Subjects were randomized into two groups to assess prophylaxis with specific GCS during flights lasting 11 h 30 min-12 h. The control group had no prophylaxis. The treatment group wore specially designed GCS (Scholl, UK), producing 14-17 mmHg of pressure at the ankle. Subjects were instructed to put on the GCS before leaving for the airport (3-4 h before the flight). The following were considered high risk for DVT or SVT: coagulation disorders, severe obesity or limited mobility caused by bone or joint problems, oncological disease during the preceding 2 years, cardiovascular disease and large varicose veins. Individuals with height exceeding 190 cm and/or weight exceeding 90 kg were excluded. Sonosite 7.5 to 13 MHz scanners with high resolution linear probes were used to examine the venous system while compressing the major veins (popliteal, femoral, and tibial). D-dimer and fibrinogen tests were conducted before flights (within 12 h) and repeated within 4 h of the end of the flight (Dade Dimertest, latex test, Behring, Germany). Digitalization was performed 90 min before flights and soon after flights (within 90 min). Patients were given suggestions for light exercise (stand up and move legs for 5 to 10 min every hour), avoid baggage between seats, and drink 100 to 150 mL of water regularly every hour. Statistical analysis was conducted using nonparametric tests and analysis of variance, considering subjects who completed the protocol as free from events. The incidence of thrombotic events (DVT, superficial thrombosis) was calculated and compared considering individuals and using intention-to-treat analysis.
			Exclusion criteria were clinical disease requiring treatment, severe bone or joint problems or limited mobility, uncontrolled diabetes, hypertension, obesity, recent thrombosis, and thrombi detected in pre-flight examination.
Hagan et al. (2008)^21^	Sydney, AUS	50 volunteers (22 pilots and 28 passengers); mean age: 24 to 71 years. GCS group: 26 participants (18 men and 6 women) on outward flight; 24 participants (17 men and 6 women) on return flight.	Twenty-six of the 50 participants wore GCS on the outward flight (group 1; 18 men and 6 women) and the other 24 wore GCS on the return flight (group 2; 17 men and 6 women). At the end of the study, 47 participants (24 in group 1 and 23 in group 2) comprised the study sample.
			Participants were assigned at random to wear low ankle pressure GCS for both outward and return flights. Random allocation was conducted by giving participants sealed envelopes containing instructions according to a computer-generated randomization sequence. The participants wore their normal clothing during the control flight (when GCS were not worn). Both groups were given exercise suggestions.
			The primary outcome was the difference in change in ankle circumference (measured before the flight and after landing) between the control group (without GCS) and treatment group (wearing GCS). Secondary outcomes included pain, discomfort, and feelings of edema in the legs, energy levels, alertness and concentration capacity, each classified one-dimensionally. The scale used was an 11-point numerical classification (NRS).
Charles et al. (2011)^13^	Wellington, NZL	20 adult participants (13 women), aged 18 to 65 years.	Participants underwent anthropometry (weight, height, and body mass index) and leg measurements to guarantee that the correct size compression stocking would be provided. Which leg the stocking would be worn on was also randomized. Participants were requested to avoid any type of strenuous physical exercise, such as running, rowing, or cycling 24 h before the USG examination. Mild to moderate physical activity such as walking or swimming was considered acceptable.
			USG was used to measure venous blood flow in the popliteal vein. Sonography of the popliteal fossa was used to position the ultrasound probe (USG Aplio XG) and blood flow was measured in the popliteal vein 0, 30, 60, and 120 minutes after the stocking had been put on. The primary outcome variable was maximum systolic velocity in the popliteal vein. Pre-planned secondary result variables were mean flow velocity, total flow volume, and cross-sectional vein area.
Olsen et al. (2019)^22^	Copenhague, DK	34 participants with mean age of 31 years (range: 25-54 years).	Controlled randomized study with a paired design, in which each participant was randomized to wear a compression stocking on just one leg. The stockings were below-the-knee length and compression class II, corresponding to 23 to 32 mmHg of pressure at the ankle. The study recruited adult men and women aged 18 to 60 years and excluded people with any type of condition that would demand GCS during flights or who had symptomatic arterial insufficiency in the lower limbs.
			Primary outcome: changes in differences in ankle circumference and leg circumference and subtraction of the circumference of the leg wearing the compression stocking, before and after the flight. Secondary outcomes were changes in differences in calf circumference before and after flights and pain and discomfort between legs with and without GCS. Pain and discomfort were also assessed.

GCS = graduated compression stockings; DVT = deep venous thrombosis; SVT = superficial venous thrombosis; PCR = polymerase chain reaction; ACE = angiotensin-converting enzyme; NRS = numerical rating scale; USG = ultrasonography.

**Table 2:** Details of the results reported by the randomized clinical trials included in the systematic review.

Author (year)	Outcome	Other results
	DVT/PTE prophylaxis	
Scurr et al. (2001)^17^	After the flights, 12 passengers who had not worn GCS developed symptomless DVT in the calf that was detected in duplex ultrasonographic examinations (10%; 95% confidence interval [CI] 4.8–16.0%]. None of the 115 passengers (95%CI 0–3.2%) who did wear GCS exhibited DVT. Four people who did wear GCS developed superficial thrombophlebitis of varicose veins (3%; 1.0-0.8%). None of the control group members developed superficial thrombophlebitis (1%; 0–3%,).	14 (7%) of the 200 participants were heterozygous for factor V Leiden (n = 11) or prothrombin (n = 4) gene mutations.
		One person who had both genetic mutations had an episode of thrombophlebitis.
		Two passengers with symptomless DVT were factor V Leiden positive.
		Complete blood count, platelet counts, and D-dimer assays did not provide prognostic information.
		Only two passengers took medications, in addition to their usual medications.
Belcaro et al. (2002)^18^	PART I: GCS group: none of the participants had DVT or superficial thromboses. Control group: 4 (2.2%) had DVT (2 popliteal and 2 in proximal tibial veins). In total, 3.35% (n = 6) subjects had a thrombotic event (p < 0.002).	PART I: Edema: After the flight, edema scores were 6.74+3.1 in the control group, compared to 2.16+1.1 in the GCS group, 2.9 times lower than in the control group (p < 0.005). Around 80% of the individuals in the control group had increases in ankle circumference and volume that was evident on examination and associated with some degree of discomfort.
	PART II: GCS group: none of the participants had DVT or superficial thromboses. Control group: 3 had DVT and 3 had superficial vein thromboses (SVT). In total, 4.2% (n = 6) subjects had a thrombotic event (p < 0.02).	PART II: Edema: After the flight, edema scores were 8.08+2.9 in the control group, compared to 2.56+1.56 in the GCS group, with p < 0.05.
Cesarone et al. (2003)^19^	PART I: None of the participants had DVT or superficial thromboses in the stockings group or the control group.	PART I: Edema: After the flight, edema scores were 6.9+2.0 in the control group, compared to 2.3+1.0, in the GCS group, three times lower than in the control group (p < 0.05). At least 89% of the individuals in the control group had increases in ankle circumference and volume that were evident on examination and associated with some degree of discomfort. Control of edema with GCS was clear, considering both parametric (circumference and volume) and nonparametric (analog scale) data.
	PART II: No DVT were observed in the stockings group. In the control group, two individuals had popliteal DVT (limited, length > 2 cm, asymptomatic) and two had superficial thrombosis (thrombi in one of the long branches of the saphenous vein, both below the knee), with p < 0.02. Four individuals (6%) in the control group exhibited thrombotic events. The incidence of DVT was 3%.	PART II: Edema: After the flight, edema scores were 7.94+2.0 in the control group, compared to 3.3+1.2 in the GCS group. In the GCS group, the hourly increase in score was 0.17 on 7-hour flights and 0.19 on 11-hour flights, compared with 0.82 and 0.63 in control group (on flights lasting 7 and 11 hours, respectively). In the GCS group, edema scores were 3.2+0.8 at enrollment and 6.2+1.4 after the 7-hour flight. In the control group, scores were 3.0+0.9 at enrollment and 9.6+0.2 at the end of the flight (p < 0.05).
	Overview: Considering DVT, the difference between control and treatment groups was significant (p < 0.05) - 2/138 = 1.44% vs. 0/138 = 0%.	
Cesarone et al. (2003)^20^	PART I: None of the 97 control group subjects who completed the study had DVT or superficial thrombosis.	PART I: Edema levels at enrollment were comparable in both groups. After the flight, edema scores were 6.4+1.3 in the control group, compared to 2.4+1.0 in the GCS group, 2.6 times lower than in the control group (p < 0.05). In the control group, 83% of the subjects had increases in ankle circumference and volume that was evident on examination and associated with some degree of discomfort.
	PART II: None of the GCS group participants had deep or superficial thrombosis. No thrombotic events were observed in any of the 71 control group members who completed the study.	PART II: Edema levels at enrollment were comparable in both groups (1.1). After the flight, edema scores were 8.9+2.0 in the control group, compared to 2.56+1.3 in the GCS group (p < 0.05). Control of edema with GCS was clear, even after 11-hour flights, considering circumference, volume, and analog scale lines. At the end of the flight, a limited quantity of edema was observed in all subjects who wore GCS. Significant edema was observed in those who did not wear GCS.
Belcaro et al. (2003)^14^	In the treatment group, just one limited, distal, DVT was observed (0.4 cm in length, soleus vein, 0.97%). In the control group, six subjects (5.8%) had DVT and there was no superficial thrombosis. The difference in DVT incidence was significant (p < 0.0025).	The intention-to-treat analysis detected 18 failures in the control group (12 lost to follow-up + six thromboses), out of 114 individuals (15.6%), vs. eight failures (7.3%) in the treatment group, with p < 0.05. All thrombotic events were totally asymptomatic. The three women who had thrombotic events in the control group and the only case in the GCS group had all been taking low dose oral contraceptives for at least 12 months before the flight.
Hagan et al. (2008)^21^	When wearing GCS, there was a reduction in edema at the ankle compared with not wearing GCS (mean difference: -0.19 cm; 95%CI, -0.33 to -0.065 cm; p = 0.012).	When wearing GCS, participants had a 60% improvement in their classification of leg pain at the end of the flight, a 50% improvement in their classification of leg discomfort, and 45% improvement in their classification of leg edema. There was also an 18% improvement in energy level, a 13% improvement in alertness, and a 12% improvement in concentration capacity.
Charles et al. (2011)^13^	At 120 minutes, peak systolic velocity was 24% greater with treatment, with a difference of 0.34 cm/s (95%CI, 0.12 to 0.56, p = 0.004). There was a reduction in the risk of VTE associated with long-haul journeys. Peak systolic velocity was 0.35 cm/s (95%CI, 0.22 to 0.49, p < 0.001) greater when stockings were worn. The overall difference between legs with and without stockings for the calf circumference measurement at 120 minutes was 7.9 mm (95%CI, –13.3 to -2.4, p = 0.011). The below-the-knee graduated compression stocking increased venous blood flow in the lower limbs during prolonged immobility in the sitting position.	The stocking was associated with a reduction in edema in the leg, ankle and calf, suggesting a potential utility for edema of the lower limbs, secondary to chronic venous insufficiency and lymphedema. Peak systolic velocity was also significantly higher at 30 and 60 minutes when wearing stockings compared with not wearing them. Mean flow velocity and total flow volume were greater at 60 and 120 minutes when wearing the stocking.
Olsen et al. (2019)^22^	There was no significant difference for DVT/PTE.	The difference in ankle circumference was 5 mm greater after the flight compared to before the flight, IQR = 0–9, p = 0.001. Legs wearing GCS had a 2 mm median reduction in ankle circumference (p = 0.004) during the flight, whereas legs without GCS had an average increase of 2 mm (p = 0.01). The difference between calf circumferences before and after the flight was 5 mm, IQR = 1–12, p < 0.001. After the flight, legs wearing compression stocking had a 3mm reduction in calf circumference (p = 0.007), whereas calf circumference of legs not wearing GCS increased by a median of 3 mm (p = 0.02).
		Pain and Discomfort: There was no significant difference in pain or discomfort in the legs after the flight compared with before the flight. Although the visual analog pain scale did not increase after the flight, there was an increase in the visual analog discomfort scale of 1 mm in legs wearing GCS (p = 0.005).

GCS = Graduated compression stockings; DVT = deep venous thrombosis; VTE = venous thromboembolism; PTE = pulmonary thromboembolism; IQR = interquartile range.

**Table 3:** Evaluation of the methodological quality of randomized clinical trials using the Consolidated Standards of Reporting Trials (CONSORT) instrument.

Author (year)	CONSORT checklist items not fulfilled
Scurr et al. (2001)^17^	Item 23: does not state registration number and name of trial registry;
	Item 24: does not state where the full trial protocol can be accessed.
Belcaro et al. (2002)^18^	Item 8a: does not describe the method used to generate the random allocation sequence
	Item 8b: does not describe type of randomization and details of any restriction;
	Item 11a and 11b: does not describe who was blinded after assignment to interventions and how;
	Item 15: does not have a table showing baseline demographic and clinical characteristics for each group;
	Item 17a: does not report the confidence interval;
	Item 20: does not state trial limitations, addressing sources of potential bias, imprecision, and, if relevant, multiplicity of analyses.
Belcaro et al. (2003)^14^	Item 1 a: not identified as randomized clinical trial in the title;
	Item 8b: does not describe type of randomization;
	Item 9: does not describe the mechanism used to implement the random allocation sequence;
	Item 10: does not state who generated the random allocation sequence, who enrolled participants, and who assigned participants to interventions;
	Item 11a and 11b: does not describe if there was blinding and who was blinded after assignment to interventions and how;
	Item 17a: does not report the confidence interval, only the p value;
	Item 24: does not state where the full trial protocol can be accessed.
Cesarone et al. (2003)^19^	Item 1a: not identified as randomized clinical trial in the title;
	Item 3a: does not describe trial design (parallel, factorial, etc.);
	Item 8b: does not state type of randomization;
	Item 9: does not describe the mechanism used to implement the random allocation sequence;
	Item 10: does not state who generated the random allocation sequence, who enrolled participants, and who assigned participants to interventions;
	Item 11a and 11b: does not state if there was blinding after assignment to interventions;
	Item 17a: does not report the confidence interval, only the p value;
	Item 17b: does not present both absolute and relative effect sizes, only p values ;
	Item 24: does not state where the full trial protocol can be accessed;
Cesarone et al. (2003)^20^	Item 3a: does not describe trial design (parallel, factorial, etc.);
	Item 8b: does not describe type of randomization and details of any restriction ;
	Item 9: does not describe the mechanism used to implement the random allocation sequence, describing any steps taken to conceal the sequence ;
	Item 10: does not state who generated the random allocation sequence, who enrolled participants, and who assigned participants to interventions.
Hagan et al. (2008)^21^	Item 9: does not describe the mechanism used to implement the random allocation sequence, describing any steps taken to conceal the sequence until interventions were assigned;
	Item 11a and 11b: not applicable to the study;
	Item 12b: does not show methods used for additional analyses, such as subgroup analyses and adjusted analyses;
	Item 14b: does not state why the trial ended or was stopped;
	Item 17b: not applicable to the study.
Charles et al. (2011)^13^	Item 3a: does not describe the trial design;
	Item 11a and 11b: does not specify blinding;
	Item 14a: does not define dates of periods of recruitment and follow-up;
	Item 23: does not state registration number and name of trial registry;
	Item 24: does not state where the full trial protocol can be accessed.
Olsen et al. (2019)^22^	None – the study fulfilled all items proposed in the CONSORT checklist.

**Table 4:** Quality of evidence for the outcomes assessed.

Outcome	N° of participants in studies	Quality of evidence (GRADE)
Edema	1,311	High
Incidence of DVT/PTE	2,022	Moderate

GRADE = Grading of Recommendations Assessment, Development and Evaluation; DVT = deep venous thrombosis; PTE = pulmonary thromboembolism.

## DISCUSSION

The objective of this review is to identify what evidence exists demonstrating the benefits of wearing GCS during long-duration flights for reducing the incidence of VTE and edema in the lower limbs. Although the incidence of DVT in the general population is low, it is increasing significantly and is even higher in patients considered at high risk, varying from 4 to 10%, so prophylaxis is therefore recommended.^17^

GCS are considered a VTE prevention method with fewer side effects than drug-based prophylaxis.^13^^,^ 18 According to some studies, there is a statistically significant effect in people who wear GCS compared to those who do not, reducing the risk of DVT by approximately 90%.^23^

The most common sign of DVT in the lower limbs is edema, observed in people who travel by air for more than 2 to 4 hours, because of restricted movement and immobility. This sign may be exacerbated in patients with diseases such as chronic venous disease, diabetic microangiopathy, and heart failure, and is very often ignored, to the extent that the passengers themselves consider it normal after spending several hours seated.^17^^-^ 20

Because of this, prophylactic measures intended to mitigate the risk of VTE have been studied, aiming to reduce the occurrence of adverse effects. The present systematic review identified studies that report reduced incidence of VTE when wearing GCS compared to when not wearing them, with quality of evidence that can be considered moderate. This non-pharmacological measure is associated with improved venous hemodynamics in the limb, as shown by measurement of peak systolic velocity, mean flow volume, and total flow volume in the popliteal vein. The studies reviewed indicate that the velocity of blood flow in the lower limbs is increased by around 0.35 cm/s.^14^

It is important to point out that interpretation of this outcome could be limited by the impossibility of blinding the ultrasound operator during examination of the popliteal vein, after allocation of participants. The results obtained nevertheless suggest increased venous blood flow in the lower limbs when wearing GCS, which is a useful strategy for reduction of VTE risk both in healthy patients and in those with chronic venous disease.^14^^,^ 17 Studies have demonstrated that formation of thrombi may be attenuated when wearing GCS, by the increased perivascular pressure and by reducing tissue factor contact, even in people at low-medium risk of DVT.^18^^,^ 21 ^,^ 24

Some studies have also reported evidence that edema is very common and is observed in almost all people who undertake long-duration flights, even those who are healthy. It was demonstrated that even small quantitative increases in ankle circumference could be associated with perceptions of lower limb edema and complaints of pain and discomfort and that wearing GCS can impede its development. Studies also indicate that edema tends to increase as the duration of the flight increases,^18^^,^ 19 ^,^ 24 although more recent studies have already found this outcome on shorter flights, of up to 3 hours’ duration, which can also increase the risk of VTE, since edema can very often supervene VTE events and is their most common sign. Recently, Olsen et al.^22^ observed a reduction in lower limb edema associated with wearing GCS in a sample of young healthy individuals. In that study, age was considered a major risk factor for edema formation and, consequently, there could be greater benefit from wearing GCS.^22^

The studies did not demonstrate significant differences in pain or discomfort on short-duration flights, comparing lower limbs wearing or not wearing GCS. They only found a discrete increase in discomfort, measured using a visual analog pain scale, of 1 mm in legs wearing GCS, which may not be a relevant fact. This discomfort may be related to the age of the participants, since they were young and may not have been used to wearing GCS, and also may be linked to the pressure exerted by the stockings, with no direct relationship with edema.^22^ In view of this, it is suggested that future studies should investigate the association between pain and discomfort related to lower limb edema on short-duration journeys.

It is valid to compare the study conducted with shorter-duration flights (3 hours) with those conducted with longer-duration flights (exceeding 5 hours). It was found that on shorter-duration flights, there was a greater difference in reduction of ankle circumference after wearing GCS and little difference in terms of improvement in pain and discomfort.^22^ On the longer-duration flights, a smaller difference was observed in reduction of ankle circumference after wearing GCS, but there was a significant difference in terms of improvement in pain and discomfort.^21^ In addition to flight duration, which exerts a direct influence on ankle circumference measurements, the age of the passengers could have contributed to these findings, since older participants might be more sensitive to formation of edema or less sensitive to the compression exerted by the GCS.^21^^,^ 22 It was also observed that regardless of whether or not GCS were worn, some studies included recommendations such as performing exercises during flights, dietary suggestions (such as encouraging drinking of water and avoidance of salty foods), and reducing the volume of on-board baggage to maintain legroom,^24^ which may also have contributed to reducing DVT and edema.

Certain limitations of the studies selected were observed, such as having been conducted in controlled research environments and with healthy participants, resulting in findings that may not be generalizable to people with venous or arterial disease or different body habits.^21^ It is therefore suggested that future studies investigate the effect of GCS on lower limb edema in passengers at increased risk of edema formation (advanced age, venous diseases) and on reduction of VTE incidence in flights lasting less than 4 hours. This statement is further reinforced by the fact that the participants in the study by Olsen et al.^22^ were young, which could limit the external validity of the results when older passengers and those with comorbidities are considered. Since age is a risk factor for lower limb edema,^25^^,^ 26 older passengers have more pronounced edema and, consequently, gain greater benefit from wearing GCS, even during short-duration flights.^22^ Moreover, it is valid to point out that assessment of VTE occurrence demands a larger number of subjects and more prolonged observations, which could possibly have made it less likely that its incidence would have been recorded in the studies selected.^18^

## CONCLUSIONS

This systematic review demonstrates that there is high quality scientific evidence for prevention of edema and moderate quality evidence for reduction of incidence of VTE by wearing GCS during long-duration flights. Edema reduction was observed after wearing GCS on flights with duration of 3 hours, suggesting that passengers with lower limb edema, experiencing pain or discomfort, would probably benefit from wearing them. However, additional randomized clinical trials are needed to investigate the effect of wearing GCS on shorter duration flights to determine whether they interfere in reduction of VTE incidence and signs such as edema, pain, and discomfort.

## References

[1] International Civil Aviation Organization The world of air transport in 2016 - Presentation of 2016 international air transport statistical results.

[2] Brenner B (2009). Prophylaxis of travel-related thrombosis in women. Thromb Res.

[3] Silverman D, Gendreau M (2009). Medical issues associated with commercial flights. Lancet.

[4] Marques MA, Panico MDB, Porto CLL, Milhomens ALM, Vieira JM (2018). Venous thromboembolism prophylaxis on flight. J Vasc Bras.

[5] Alvares F, Pádua AI, Terra J (2003). Tromboembolismo pulmonar: Diagnóstico e tratamento. Medicina (B Aires).

[6] Volschan A, Caramelli B, Gottschall CAM (2004). Diretriz de embolia pulmonar. Arq Bras Cardiol.

[7] Brandã RA, Velasco IT, Brandão RA (2019). Trombose venosa profunda.. Medicina de emergência: abordagem prática..

[8] Cervantes J, Rojas G (2005). Virchow’s legacy: deep vein thrombosis and pulmonary embolism. World J Surg.

[9] López JA, Kearon CAYYL, Lee AY (2004). Deep venous thrombosis. Hematology (Am Soc Hematol Educ Program).

[10] Kuipers S, Cannegieter SC, Middeldorp S, Robyn L, Büller HR, Rosendaal FR (2007). The absolute risk of venous thrombosis after air travel: a cohort study of 8,755 employees of international organisations. PLoS Med.

[11] Chandra D, Parisini E, Mozaffarian D (2009). Meta-analysis: travel and risk for venous thromboembolism. Ann Intern Med.

[12] Lapostolle F, Surget V, Borron SW (2001). Severe pulmonary embolism associated with air travel. N Engl J Med.

[13] Charles T, Mackintosh D, Healy B, Perrin K, Weatherall M, Beasley R (2011). Merino wool graduated compression stocking increases lower limb venous blood flow: A randomized controlled trial. Adv Ther.

[14] Belcaro G, Cesarone MR, Nicolaides AN (2003). Prevention of Venous Thrombosis with Elastic Stockings During Long-Haul Flights: The LONFLIT 5 JAP Study. Clin Appl Thromb Hemost.

[15] Schulz KF, Altman DGMD, Moher D, CONSORT Group (2010). CONSORT 2010 Statement: updated guidelines for reporting parallel group randomised trials. Trials.

[16] Brasil (2014). Diretrizes Metodológicas. Sistema GRADE - manual de graduação da qualidade da evidência e força da recomendação para tomada de decisão em saúde.

[17] Scurr JH, Machin SJ, Bailey-King S, Mackie IJ, Mcdonald S, Smith PDC (2001). Frequency and prevention of symptomless deep-vein thrombosis in long-haul flights: a randomised trial. Lancet.

[18] Belcaro G, Cesarone MR, Shah SSG (2002). Prevention of edema, flight microangiopathy and venous thrombosis in long flights with elastic stockings. A randomized trial the LONFLIT 4 concorde Edema-SSL study. Angiology.

[19] Cesarone MR, Belcaro G, Errichi BM (2003). The LONFLIT4-concorde deep venous thrombosis and edema study: prevention with travel stockings. Angiology.

[20] Cesarone MR, Belcaro G, Brandolini R (2003). The LONFLIT4-Venoruton study: a randomized trial-prophylaxis of flight-edema in venous patients. Angiology.

[21] Hagan MJ, Lambert SM (2008). A randomised crossover study of low-ankle-pressure graduated-compression tights in reducing flight-induced ankle oedema. Med J Aust.

[22] Olsen JHH, Öberg S, Rosenberg J (2019). The effect of compression stocking on leg edema and discomfort during a 3-hour flight: a randomized controlled trial. Eur J Intern Med.

[23] Clarke MJ, Broderick C, Hopewell S, Juszczak E, Eisinga A (2016). Compression stockings for preventing deep vein thrombosis in airline passengers. Cochrane Database Syst Rev.

[24] Cesarone MR, Belcaro G, Nicolaides AN (2003). The LONFLIT4-Concorde-Sigvaris Traveno Stockings in Long Flights (EcoTraS) study: a randomized trial. Angiology.

[25] Evans CJ, Fowkes FG, Ruckley CV, Lee AJ (1999). Prevalence of varicose veins and chronic venous insufficiency in men and women in the general population: Edinburgh Vein Study. J Epidemiol Community Health.

[26] Criqui MH, Jamosmos M, Fronek A (2003). Chronic venous disease in an ethnically diverse population: the San Diego Population Study. Am J Epidemiol.

