# Supplementary material for: Graduated compression stockings as a prophylactic measure in venous thromboembolism and edema of lower limbs triggered by air travel: a systematic review of clinical trials
Source: J Vasc Bras. 2021 May 10;20:e20200164. doi: 10.1590/1677-5449.200164 (PMC8147891; doi:10.1590/1677-5449.200164)
Supplement: Translation

# Translation: Meias elásticas de compressão graduada como medida profilática de tromboembolismo venoso e edema de membros inferiores desencadeados por viagens aéreas: uma revisão sistemática de ensaios clínicos

**Authors:** Lorenna Ferreira da Silva, Melissa Stephane Roberta Porto, Albert Bacelar de Sousa, Katia de Miranda Avena

**Affiliations:** 1 Centro Universitário UNIFTC, Salvador, BA, Brasil.

**Keywords:** embolia pulmonar, viagens aéreas, meias compressivas, trombose venosa, prevenção de doenças, segurança do paciente

## Abstract

Com o aumento da duração e frequência das viagens aéreas, observou-se um aumento da prevalência de tromboembolismo venoso nos passageiros. Este estudo avaliou a eficácia do uso de meias elásticas de compressão graduada para a prevenção de tromboembolismo venoso desencadeado por viagens aéreas com duração maior que 3 horas de voo. Trata-se de uma revisão sistemática de ensaios clínicos. A qualidade metodológica dos estudos e o nível de evidência científica foram avaliados pelo *Consolidated Standards of Reporting Trials* e *Grading of Recommendations Assessment, Development and Evaluation*. Foram identificados 34 artigos, entretanto apenas oito atenderam aos critérios de elegibilidade. Os desfechos incidência de tromboembolismo venoso e edema foram avaliados em 2.022 e 1.311 passageiros, respectivamente. Os estudos demonstraram evidências de alta qualidade para a prevenção de edema e de moderada qualidade para a redução da incidência de tromboembolismo venoso com o uso de meias elásticas de compressão graduada durante viagens aéreas.

## INTRODUÇÃO

Um dos meios de viagem mais utilizados mundialmente é o transporte aéreo, o qual conduz bilhões de pessoas por ano. Na última década, o número de viagens aéreas vem aumentando constantemente pelo incremento da quantidade de voos disponíveis; portanto, mais pessoas estão sendo transportadas. Estudos demonstram que, por ano, 3,8 bilhões de pessoas viajam de avião e, dessas, 300 milhões fazem voos de duração prolongada^1^^-^ 3, aumentando, assim, o risco de doenças associadas a viagens aéreas com duração maior que 3 horas, sendo a trombose venosa profunda (TVP) e o tromboembolismo pulmonar (TEP) as principais^4^.

A TVP e o TEP fazem parte do espectro da mesma doença: o tromboembolismo venoso (TEV). A TVP ocorre principalmente nos membros inferiores (MMII) a partir de coágulos que se formam no sistema venoso profundo. Quando um desses coágulos se desprende, alcança as câmaras cardíacas direita e, posteriormente, a circulação pulmonar com redução ou cessação do fluxo sanguíneo em segmentos pulmonares, caracterizando, assim, o TEP, e podendo gerar um quadro de dispneia, dor pleurítica e hemoptise. O TEV pode levar também a complicações crônicas, como hipertensão pulmonar e síndrome pós-trombótica^5^^-^ 7.

Segundo a tríade de Virchow^5^, três fatores podem contribuir, isoladamente ou em conjunto, para o desenvolvimento de TEV: estase do fluxo sanguíneo, hipercoagulabilidade e lesão endotelial^8^^,^ 9. A ocorrência mundial de TEP relacionado a viagens aéreas ainda é relativamente baixa, com uma incidência de 3,2 casos/1.000 pessoas/ano. Essa incidência é cerca de 3,2 vezes maior que na população saudável que não viaja por transporte aéreo^10^. Além disso, o risco de desenvolvimento de TEP também pode duplicar em voos com mais de 8 horas de duração e aumenta 26% a cada 2 horas acrescidas na duração da viagem^11^. Considerando o risco de TEP grave, a incidência é de aproximadamente 0,4 casos por milhão de passageiros^12^.

O desenvolvimento de TEV durante o voo tem sido associado principalmente à imobilidade temporária de MMII pela permanência prolongada no assento, o que aumenta a estase venosa e reduz o fluxo sanguíneo em veias profundas e superficiais dos MMII^13^. Outros fatores incluem doenças ou condições que favoreçam a formação de TVP ou TEP, como história de TEV e trombofilias, entre outras^5^.

As meias elásticas de compressão graduada (MECG) representam um método não farmacológico para a redução do risco de TEV nos casos em que há imobilidade prolongada, pois reduzem a estase venosa por aumentar o fluxo sanguíneo venoso nos MMII^13^. Tendo em vista o aumento da frequência das viagens aéreas e, consequentemente, o risco aumentado para o desenvolvimento de TEV, estudos vêm buscando encontrar medidas profiláticas que sejam eficientes e seguras. Nesse contexto, uma das possíveis estratégias para a prevenção dessas doenças é o uso de MECG durante o voo.

A ocorrência do edema também é um aspecto relevante em voos de longa duração, afetando tanto pacientes com doenças venosas quanto indivíduos saudáveis. Estudos vêm demonstrando que o edema é observado em quase todos os indivíduos que voam por mais de 7 horas, chegando a ocorrer em cerca de 97% dos passageiros, sendo mais evidente em pacientes com doença venosa crônica^14^.

Diante do exposto, o objetivo deste artigo é verificar a existência de evidência científica quanto ao uso de MECG durante viagens aéreas como medida profilática para TEV e formação de edema de MMII.

## MÉTODOS

Foi realizada uma revisão sistemática da literatura seguindo esta sequência lógica: 1) elaboração da pergunta de investigação, com base na estratégia PICOT (*Patient or Problem, Intervention, Control or Comparison, Outcomes*); 2) identificação das bases de dados para a seleção dos estudos; 3) definição de critérios de inclusão e exclusão; e 4) avaliação da qualidade metodológica dos artigos identificados.

Como se trata de um estudo a partir de dados secundários provenientes da literatura, não envolvendo experimentação primária em seres humanos, foi dispensada a submissão ao Comitê de Ética em Pesquisa.

### Estratégia de busca

A pergunta de investigação que norteou a estratégia de busca foi: “o uso de MECG durante viagens aéreas é eficaz como medida profilática para o desenvolvimento de TEV e edema de MMII?”. O levantamento dos artigos científicos foi realizado nas plataformas PubMed e Biblioteca Virtual de Saúde. Com base nos descritores em saúde (DECs e MeSH), foram estabelecidas como palavras-chave: “*Pulmonary embolism*”, “*Air travel*”, “*Stockings, Compression*” e seus correlatos na língua portuguesa. O cruzamento entre as palavras-chave foi realizado da seguinte forma na PubMed: “*Pulmonary embolism*” AND “*Air travel*” AND “*Stockings, Compression*”. Essa estratégia de busca foi adaptada para a Biblioteca Virtual de Saúde. A última atualização ocorreu em 30 de abril de 2020.

De forma intencional, foram inicialmente empregados termos mais amplos no intuito de identificar uma maior quantidade de estudos e, assim, minimizar a chance de que algum artigo importante pudesse ser excluído desse levantamento. Portanto, foi realizada a busca manual de artigos através das listas de referência dos estudos primários incluídos, com o intuito de identificar estudos relevantes que atendessem aos critérios de elegibilidade, mas que, por algum motivo, não tivessem sido identificados através da estratégia de busca definida.

### Critérios de elegibilidade

Não foram impostas restrições quanto à data de publicação dos estudos nem quanto ao idioma das publicações. Por se tratar de uma revisão sistemática de ensaios clínicos randomizados, foram excluídos livros, capítulos de livros, editoriais, revisões narrativas e sistemáticas, entre outros formatos de texto que não tenham passado por rigoroso processo de avaliação por pares, como ocorre com os artigos científicos. Além disso, foram excluídos os textos encontrados em duplicidade pela indexação em mais de uma base de dados.

### Coleta de dados, classificação e avaliação da qualidade dos estudos

A incidência de TEV assintomático/sintomático e a presença de edemas nos MMII com o uso ou não de MECG durante viagens aéreas foram consideradas como variáveis de interesse a serem revisadas por este estudo. O levantamento dos dados bibliográficos foi realizado de 1 de fevereiro a 30 de abril de 2020. Após a identificação dos estudos, foi realizada a avaliação crítica deles. Essa etapa consistiu na leitura e análise dos títulos e resumos dos estudos identificados, sendo realizada por dois revisores independentes. Nesse momento, foram excluídos os estudos que não atendiam à proposta desta revisão sistemática. Após a triagem inicial, a etapa seguinte consistiu na leitura integral dos estudos, o que possibilitou que alguns textos fossem excluídos por não serem considerados confiáveis (por questões metodológicas, de randomização, conflitos de interesse, entre outros fatores). Por último, os estudos foram organizados em uma tabela no intuito de apresentar suas principais informações, facilitando a análise descritiva e crítica dos resultados obtidos pelos autores.

A qualidade metodológica dos estudos foi avaliada através do *Consolidated Standards of Reporting Trials* (CONSORT)^15^, uma *checklist* composta por 25 itens, permitindo que seja possível entender os métodos, a análise e a validade dos resultados apresentados pelos ensaios clínicos randomizados incluídos nesta revisão. Para classificar a qualidade da evidência científica e a força das recomendações, foi utilizado o sistema *Grading of Recommendations Assessment, Development and Evaluation* (GRADE)^16^. No sistema GRADE, a qualidade da evidência é avaliada para cada desfecho analisado, sendo classificada em quatro níveis: alto, moderado, baixo e muito baixo. Para determinar o nível da evidência e definir sua classificação, foram considerados seguintes fatores: delineamento do estudo; limitações metodológicas (risco de viés); inconsistências; evidências indiretas; imprecisão; viés de publicação; magnitude de efeito; gradiente dose-resposta; e fatores de confusão residuais.

## RESULTADOS

Foram identificados 34 artigos, dos quais 14 foram excluídos após a leitura dos títulos. Dos 20 artigos restantes, após a leitura dos resumos, foi excluído outro por não atender aos critérios de elegibilidade. Sendo assim, 19 artigos foram selecionados para leitura integral dos textos. Após essa leitura, outros 11 textos foram excluídos por também não atenderem aos critérios de elegibilidade. Dessa forma, a amostra final foi composta por oito artigos.

A Figura 1 apresenta o fluxograma do número de artigos encontrados e selecionados após a aplicação dos critérios de inclusão e exclusão. Após a seleção dos artigos, as características básicas dos oito estudos elegíveis foram listadas nas Tabelas 1 e 2, facilitando a análise descritiva e crítica dos resultados obtidos pelos autores. A avaliação da qualidade metodológica dos estudos realizada com o instrumento CONSORT é apresentada na Tabela 3, considerando os métodos, a análise e a validade dos resultados apresentados pelos ensaios clínicos randomizados incluídos nesta revisão. A avaliação da qualidade da evidência do desfecho avaliado, considerando os estudos incluídos nesta revisão sistemática, é apresentada na Tabela 4. Após análise pelo sistema GRADE, observou-se que há confiança alta/moderada nos efeitos estimados; entretanto, futuros ensaios clínicos randomizados poderão ser capazes de modificar a confiança na estimativa de efeito.

**Figura 1:** Fluxograma de seleção dos artigos.

**Tabela 1:** Sumário das principais características descritivas dos estudos incluídos.

Autor (ano)	Local do estudo	Amostra detalhada	Método
Scurr et al. (2001)^17^	Londres, UK	231 passageiros (89 homens e 142 mulheres), com idade superior a 50 anos, sem história de problemas tromboembólicos que pretendiam viajar na classe econômica por pelo menos 8 h em 6 semanas.	Alocação aleatória dos passageiros em dois grupos: um grupo usava MECG classe I (padrão alemão de compressão Hohenstein 20-30 mmHg) abaixo do joelho e o outro grupo, não.
		31 foram excluídos por problemas de saúde, mudanças de planos de viagem ou incapacidades de manter compromissos.	Foi mensurada a duração de fluxo por cor ou sonografia com Doppler pulsado. A USG dúplex foi utilizada para avaliar veias profundas antes e depois da viagem por um técnico que desconhecia o grupo ao qual o voluntário havia sido randomizado. A presença de doença venosa atual ou trombose anterior foi avaliada a partir da imagem em modo B, mapeamento de cor de fluxo e avaliação de veias durante a imagem em modo B. Amostras de sangue foram analisadas para duas mutações genéticas comuns específicas, fator V Leiden (FVL) e protrombina G20210A (PGM) a partir da técnica de PCR, que predispõe ao tromboembolismo venoso. Um ensaio D-dímero sensível foi utilizado para rastrear o desenvolvimento de trombose recente.
			Nos primeiros 30 voluntários, o exame de USG foi realizado 2 semanas antes da viagem e novamente dentro de 2 dias do início do primeiro voo para fornecer um controle do intervalo em que a ocorrência de TVP espontânea poderia ser avaliado nessa população.
Belcaro et al. (2002)^18^	Itália, ITA	657 passageiros, com médio risco para TVP de ambos os sexos.	PARTE I (voos de 7-8 h): 372 participantes randomizados em dois grupos: grupo controle sem profilaxia e grupo tratamento com MECG projetadas (Flights Socks, Scholl produzindo 14-17 mmHg de pressão no tornozelo). Critérios de alto risco: episódios anteriores de TVP ou trombose venosa superficial, distúrbio de coagulação, obesidade severa, limitação da mobilidade devido a problemas ósseos ou articulares, doença neoplásica nos 2 anos anteriores, doença cardiovascular ou varizes grandes. Foram excluídos indivíduos com altura superior a 190 cm e peso superior a 90 kg. Protocolo de varredura por USG (antes e depois do voo): *scanners* sonosite de 7,5 a 13 MHz com sonda linear de alta resolução foram utilizados para avaliar o sistema venoso comprimindo as principais veias (poplítea, femorais). Sugestões para os passageiros — realizar exercícios leves, como caminhar; beber água e evitar alimentos salgados; evitar excesso de bagagens que restrinja o movimento das pernas — foram dadas a ambos os grupos. O edema foi baseado nos testes de edema, circunferência de tornozelo e escore de desconforto.
		PARTE I: Grupo MECG: 101 homens e 78 mulheres, idade de 49,0±7;0 anos. Grupo controle: 98 homens e 81 mulheres, idade de 48,4±7,3 anos.	PARTE II (voos de 11-12 h): 285 participantes randomizados em dois grupos: o grupo controle sem profilaxia e o grupo de tratamento com MECG abaixo do joelho (Flights Socks, Scholl, UK, produzindo 14-17 mmHg de pressão no tornozelo). Os mesmos procedimentos e critérios adotados na parte I foram utilizados.
		PARTE II: Grupo MECG: 89 homens e 53 mulheres, idade de 48,0±8,0 anos. Grupo controle: 87 homens e 56 mulheres, idade de 47,0±8,0 anos.	
Cesarone et al. (2003)^19^	Itália, ITA	284 passageiros de baixo-médio risco de ambos os sexos nas partes I e II.	PARTE I (voos de 7-8 h): 144 participantes randomizados em dois grupos: o grupo controle não teve profilaxia e o grupo tratamento utilizou MECG (Kendall, Travel Socks, Tyco Healthcare, Mansfield, EUA) produzindo 20-30 mmHg de pressão ao tornozelo, com pressão decrescente no meio da panturrilha. Sugestões em relação a atividades de bordo foram dadas a ambos os grupos de passageiros (exercícios leves, caminhadas, beber água, evitar alimentos salgados e evitar bagagens excessivas que restrinjam o movimento das pernas). Protocolo de varredura por ultrassom (antes/depois dos voos): *scanners* Sonosite com uma sonda linear de alta resolução e 7,5-13 MHz (Sonosite, Bothell, EUA) foram utilizados na avaliação do sistema venoso através da compressão das principais veias (femorais, poplítea e tibial). Edema avaliado através do escore de proposto por Valentino e Irvine, que tem como base a combinação de dados: circunferência do tornozelo (cm) e volume (mL), combinados com avaliações subjetivas de edema e desconforto.
		PARTE I: Grupo MECG: idade de 46±8,0 anos, 37 homens e 35 mulheres. Grupo controle: idade de 47,0±5,0 anos, 38 homens e 34 mulheres.	PARTE II (voos de 11-12 h): 132 participantes randomizados em dois grupos: o grupo controle não teve profilaxia e o grupo tratamento usou MECG Kendall, abaixo do joelho. 30 participantes com microangiopatia associada ao edema (10 com diabetes, 10 com insuficiência venosa crônica e hipertensão venosa e 10 em tratamento anti-hipertensivo com inibidores da ECA para hipertensão) foram incluídos para avaliar os efeitos das MECG durante os voos. Os mesmos procedimentos e critérios adotados na parte I foram utilizados.
		PARTE II: Grupo MECG: idade de 47,0±5,0 anos, 34 homens e 30 mulheres. Grupo Controle: idade de 46,9±4,0 anos, 34 homens e 32 mulheres.	
Cesarone et al. (2003)^20^	Londres, UK	PARTE I: 266 indivíduos de baixo-médio risco para TVP, sendo 55 excluídos por problemas não médicos relacionados a viagens.	PARTE I (voos de 7-8h): 211 participantes randomizados em dois grupos: o grupo controle não tinha profilaxia e o grupo tratamento usou MECG abaixo do joelho (Traveno, Sigvaris, Brasil), produzindo 12-18 mmHg de pressão no tornozelo. Critérios de risco para TVP: episódios anteriores de TVP ou trombose superficial, distúrbios da coagulação, obesidade grave, limitação de mobilidade devido a problemas ósseos ou articulares, doenças neoplásicas nos 2 anos anteriores, doença cardiovascular ou grandes varizes. Indivíduos com altura maior que 190 cm e com mais de 90 kg foram excluídos. Protocolo de digitalização por ultrassom (antes/depois dos voos): *scanners* Sonosite com uma sonda linear de alta resolução e 7,5-13 MHz (Sonosite, Bothell WA, EUA) foram utilizados na avaliação do sistema venoso através da compressão das principais veias (femorais, poplítea e tibial). Sugestões para os passageiros — realizar exercícios leves, como caminhar; beber água e evitar alimentos salgados; evitar excesso de bagagens que restrinja o movimento das pernas — foram dadas a ambos os grupos. O edema foi avaliado pelo teste de edema, circunferência do tornozelo, edema e escore de desconforto. O escore combinado de edema foi desenvolvido, baseado na avaliação combinada de dados paramétricos como o teste de edema, variações na circunferência do tornozelo (cm), medidas de volume (mL ou percentual do volume), combinado com avaliações subjetivas de edema e desconforto (de 0 a 10) definido diretamente pelos sujeitos antes e depois dos voos.
		PARTE II: 200 indivíduos de baixo-médio risco, excluindo 35 por motivos não médicos.	PARTE II (voos de 11-12 h): 165 participantes restantes randomizados em dois grupos: o grupo controle não teve profilaxia e o grupo tratamento usou MECG Traveno abaixo do joelho. Os mesmos procedimentos e critérios adotados na parte I foram utilizados.
Belcaro et al. (2003)^14^	Itália, ITA	300 indivíduos com alto risco de TVP foram contatados e pré-incluídos; 76 foram excluídos com base em várias considerações e foram randomizados em dois grupos (MECG, n = 110, *versus* controles, n = 114) para avaliar a profilaxia com MECG abaixo do joelho.	Os sujeitos foram randomizados em dois grupos para avaliar a profilaxia com MECG específicas em 11 h 30 min-12 h de voos. O grupo controle não teve a profilaxia. O grupo de tratamento utilizou MECG especificamente projetadas (Scholl, UK), produzindo 14-17 mmHg de pressão no tornozelo. Os sujeitos foram orientados a usar as MECG antes de ir para o aeroporto (3-4 h antes do voo). São considerados de alto risco para TVP ou TVS: distúrbios de coagulação, obesidade grave ou limitação da mobilidade devido a osso ou articulação, problemas neoplásicos no período anterior de 2 anos, doença cardiovascular e grandes varizes. Indivíduos com altura maior que 190 cm e mais de 90 kg foram excluídos. Os *scanners* Sonosite com uma frequência de 7,5 a 13 MHz, sonda linear de alta resolução, foram utilizados para estudar o sistema venoso por compressão das principais veias (poplítea, femoral e tibial). Os testes D-dímero fibrinogênio foram realizados antes (dentro de 12 h) dos voos e repetidos dentro de 4 h após o voo (Dade Dimertest, Teste de látex, Behring, Alemanha). A digitalização foi realizada em 90 min antes dos voos e logo após os voos (dentro de 90 min). Foram dadas sugestões de exercícios leves (ficar em pé e movimentar as pernas por 5 a 10 min a cada hora), evitar bagagem entre assentos, beber regularmente 100 a 150 mL de água a cada hora. A análise estatística foi realizada usando testes não paramétricos e análise de variância considerando livre de eventos sujeitos que completam o protocolo. A incidência de eventos trombóticos (TVP, trombose superficial) foi calculada e comparada considerando indivíduos e usando a análise com a intenção de tratar.
			Os critérios de exclusão foram doença clínica que exigisse tratamento, problemas ósseos/articulares graves ou mobilidade limitada, diabetes não controlado, hipertensão, obesidade, trombose recente e presença de trombos no exame anterior ao voo.
Hagan et al. (2008)^21^	Sydney, AUS	50 voluntários (22 pilotos e 28 passageiros); média de idade: 24 a 71 anos. Grupo MECG: 26 participantes (18 homens e 6 mulheres) na viagem de ida; 24 participantes (17 homens e 6 mulheres) no voo de volta.	Dos 50 participantes, 26 usavam MECG em voo de ida (grupo 1; 18 homens e 6 mulheres) e os 24 restantes usavam MECG em seu voo de volta (grupo 2; 17 homens e 6 mulheres). Ao final, 47 participantes (24 no grupo 1 e 23 no grupo 2) compuseram o estudo.
			Os participantes foram designados aleatoriamente para usar MECG de baixa pressão no tornozelo, tanto no voo de ida quanto no retorno. A alocação aleatória foi alcançada dando aos participantes um envelope lacrado com instruções de acordo com uma sequência de randomização gerada por computador. Durante o voo de controle (quando os MECG não foram usados), os participantes usavam suas roupas habituais. Sugestões para exercícios durante o voo foram dadas aos dois grupos.
			O desfecho primário foi a diferença na alteração da circunferência do tornozelo (medidas tomadas antes do voo e após o pouso) entre o grupo controle (sem MECG) e grupo de tratamento (MECG). Os desfechos secundários incluíram dor, desconforto e sensação de edema nas pernas, níveis de energia, estado de alerta e capacidade de concentração, cada um classificado de forma unidimensional. A escala utilizada foi de classificação numérica de 11 pontos (NRS).
Charles et al. (2011)^13^	Wellington, NZL	20 participantes adultos (13 mulheres), com idade entre 18 e 65 anos de idade.	Os participantes foram submetidos a medidas de antropometria (peso, altura e índice de massa corporal) e medidas das pernas utilizadas para garantir que uma meia de compressão de tamanho correto fosse fornecida. A perna em que a meia seria usada também foi randomizada. Os participantes foram solicitados de abster-se de realizar qualquer exercício físico extenuante, como corrida, remo ou ciclismo 24 h antes do exame de USG; atividade física leve a moderada, como caminhada ou natação, foi considerada aceitável.
			Foi utilizado USG para a medição do fluxo sanguíneo venoso na veia poplítea. Foi utilizado sonógrafo na fossa poplítea para posicionar a sonda de ultrassom (USG Aplio XG), que mensurou fluxo sanguíneo da veia poplítea 0, 30, 60 e 120 minutos após a aplicação da meia. A variável de desfecho primário foi a velocidade sistólica máxima na veia poplítea. As variáveis de resultado secundário pré-planejadas foram velocidade média do fluxo, fluxo total de volume e área transversal da veia.
Olsen et al. (2019)^22^	Copenhague, DK	34 participantes com média de idade de 31 anos (faixa 25-54 anos).	Estudo controlado randomizado, com *design* pareado, em que cada participante foi randomizado para usar uma meia de compressão em apenas uma perna, com comprimento abaixo do joelho e classe II de compressão correspondendo a 23 a 32 mmHg de compressão no tornozelo. Foram incluídos homens e mulheres, adultos de 18 a 60 anos, e excluídos aqueles com qualquer condição que exija MECG durante os voos ou insuficiência arterial sintomática nas extremidades inferiores.
			Desfecho primário: mudanças nas diferenças de circunferência do tornozelo com a circunferência da perna e subtração da circunferência da perna com meia de compressão, antes e após o voo. Desfechos secundários: mudanças nas diferenças antes e após o voo da circunferência da panturrilha e dor e desconforto entre as pernas tratados com e sem MECG. Além disso, foram avaliados dor e desconforto.

MECG = meias elásticas de compressão graduada; TVP = trombose venosa profunda; TVS = trombose venosa superficial; PCR = reação em cadeia de polimerase (*polymerase chain reaction)*; ECA = enzima conversora da angiotensina; NRS = escala de classificação numérica; USG = ultrassonografia.

**Tabela 2:** Detalhamento dos resultados obtidos pelos ensaios clínicos randomizados incluídos na revisão sistemática.

Autor (ano)	Desfecho	Outros resultados
	Profilaxia TVP/TEP	
Scurr et al. (2001)^17^	Após a viagem aérea, 12 passageiros que não usavam MECG desenvolveram TVP sem sinais na panturrilha, que foram detectados no exame ultrassonográfico dúplex [10%; intervalo de confiança (IC) 95% 4,8–16,0%]. Nenhum dos 115 passageiros (IC 0–3,2%) que usavam MECG apresentaram TVP. Quatro pessoas que usavam MECG desenvolveram tromboflebite superficial em varizes (3%; 1,0-0,8%). Ninguém do grupo controle desenvolveu tromboflebite superficial (1%; 0–3%).	14 (7%) dos 200 participantes eram heterozigotos para o fator V mutação do gene Leiden (n = 11) ou protrombina (n = 4).
		Uma pessoa teve ambas as mutações genéticas e teve um episódio de tromboflebite.
		Dois passageiros com TVP sem sinais foram fator V positivo de Leiden.
		O hemograma completo, plaquetas contagem e ensaios com D-dímero não forneceram informações prognósticas.
		Só dois passageiros usaram medicamentos, além dos habituais.
Belcaro et al. (2002)^18^	PARTE I: Grupo MECG: nenhum participante apresentou TVP ou tromboses superficiais. Grupo controle: 4 (2,2%) apresentaram TVP (2 poplítea e 2 em veias tibiais proximais). No total, 3,35% (n = 6) sujeitos apresentaram um evento trombótico (p < 0,002).	PARTE I: Edema: Após o voo, houve uma pontuação de 6,74+3,1 no grupo controle, enquanto no grupo MECG a pontuação foi de 2,16+1,1, 2,9 vezes menor que no grupo controle (p < 0,005). Cerca de 80% dos indivíduos no grupo controle tiveram um aumento na circunferência e volume do tornozelo, evidente na inspeção e associado a algum grau de desconforto.
	PARTE II: Grupo MECG: nenhum apresentou TVP ou tromboses superficiais. Grupo controle: 3 apresentaram TVP e 3 tromboses de veias superficiais (SVT). No total, 4,2% (n = 6) sujeitos apresentaram um evento trombótico (p < 0,02).	PARTE II: Edema: Após o voo, houve uma pontuação de 8,08+2,9 nos controles, enquanto no grupo MECG o escore foi de 2,56+1,56, com p < 0,05.
Cesarone et al. (2003)^19^	PARTE I: No grupo meia e no grupo controle, nenhum dos participantes tiveram TVP ou tromboses superficiais.	PARTE I: Edema: Depois do voo, houve uma pontuação de 6,9+2,0 no grupo controle. No grupo MECG, a pontuação foi de 2,3+1,0, três vezes menor do que no grupo controle (p < 0,05). Pelo menos 89% dos indivíduos no grupo controle tiveram aumento na circunferência do tornozelo e volume evidente na inspeção e associado a algum desconforto. O controle do edema com MECG ficou claro considerando os dados paramétricos (circunferência e volume) e não paramétrico (escala analógica).
	PARTE II: No grupo meia, não foi observada TVP. No grupo controle, dois indivíduos tiveram TVP poplítea (limitado, > 2 cm de comprimento, assintomático) e dois tiveram trombose superficial (trombos em um dos ramos longos veia safena, ambos abaixo do joelho), com p < 0,02. Quatro indivíduos (6%) no grupo controle apresentaram evento trombótico. A incidência de TVP foi 3%.	PARTE II: Edema: Após o voo, houve uma pontuação de 7,94+2,0 no grupo controle, enquanto no grupo tratamento o escore foi de 3,3+1,2. No grupo MECG, o aumento na pontuação por hora foi de 0,17 nos voos de 7 horas e 0,19 em voos de 11 horas em comparação com 0,82 e 0,63 no grupo controle (em voos de 7 e 11 horas, respectivamente). No grupo MECG, o escore de edema foi de 3,2+0,8 na inclusão e 6,2+1,4 ao final das 7 horas de voo. Já no grupo controle, foi de 3,0+0,9 na inclusão e 9,6+0,2 na conclusão do voo (p < 0,05).
	Visão global: Considerando a TVP, a diferença entre o grupo controle e tratamento foi significativa (p < 0,05) - 2/138 = 1,44% *versus* 0/138 = 0%.	
Cesarone et al. (2003)^20^	PARTE I: Dos 97 sujeitos que completaram o estudo, nenhum apresentou TVP ou trombose superficial entre controles.	PARTE I: O nível de edema na inclusão foi comparável nos dois grupos. Após o voo, houve uma pontuação de 6,4+1,3 no grupo controle, enquanto no grupo MECG foi de 2,4+1,0, 2,6 vezes menor que no grupo controle (p < 0,05). No grupo controle, 83% dos sujeitos tiveram aumento evidente na circunferência do tornozelo e volume claramente visível na inspeção e associado ao desconforto.
	PARTE II: No grupo MECG, nenhum participante apresentou trombose profunda ou superficial. No grupo controle, dos 71 indivíduos que completaram o estudo, nenhum evento trombótico foi observado.	PARTE II: O nível de edema na inclusão foi comparável nos dois grupos (1,1). Após o voo, houve uma pontuação de 8,9+2,0 nos controles, enquanto no grupo MECG foi de 2,56+1,3 (p < 0,05). O controle do edema com as MECG, mesmo após 11 horas de voo, foi evidente, considerando circunferência, volume e linhas de escala analógicas. No final do voo, quantidade limitada de edema foi observada em todos os sujeitos que usavam MECG. Edemas significativos foram observados em quem não usou MECG.
Belcaro et al. (2003)^14^	No grupo tratamento, apenas uma limitada TVP (0,4 cm de comprimento) distal (veia solear) foi observada (0,97%). No grupo controle, seis sujeitos (5,8%) apresentavam TVP, e não houve trombose superficial. A diferença na incidência de TVP foi significativa (p < 0,0025).	A análise de intenção de tratar detectou 18 falhas no grupo controle (12 perdas no seguimento + seis tromboses) de 114 indivíduos (15,6%) *versus* oito falhas (7,3%) no grupo de tratamento, com p < 0,05. Todos os eventos trombóticos foram totalmente assintomáticos. As três mulheres que tiveram evento trombótico (no grupo controle) e o único caso no grupo MECG estavam tomando contraceptivos orais de baixa dose por pelo menos 12 meses antes do voo.
Hagan et al. (2008)^21^	Ao usar MECG, houve diminuição no edema do tornozelo em comparação com não usar MECG (diferença média, -0,19 cm; IC95%, -0,33 a -0,065 cm; p = 0,012).	Ao usar MECG, os participantes tinham 60% de melhora na classificação da dor na perna ao final do voo, uma melhoria de 50% na sua classificação de desconforto nas pernas e 45% de melhoria na classificação de edema das pernas. Houve também uma melhoria de 18% no nível de energia, uma melhoria de 13% em seu nível de alerta e uma melhoria de 12% na capacidade de concentração.
Charles et al. (2011)^13^	A 120 minutos, o pico da velocidade sistólica foi 24% maior com o tratamento, com diferença de 0,34 cm/s (IC95%, 0,12 a 0,56, p = 0,004). Houve redução do risco de TEV associado a viagens de longa distância. O pico velocidade sistólica foi de 0,35 cm/s (IC95%, 0,22 a 0,49, p < 0,001) maior que com o uso de meia. A diferença geral para medição de 120 minutos na circunferência da panturrilha entre a perna com e sem meia foi de –7,9 mm (IC95%, –13,3 a -2,4, p = 0,011). A meia de compressão graduada abaixo do joelho aumentou o fluxo sanguíneo venoso dos membros inferiores durante a imobilidade prolongada na posição sentada.	A meia foi associada a redução no edema da perna, tornozelo e panturrilha, o que sugere uma utilidade potencial no edema de membros inferiores, secundário a insuficiência venosa crônica e linfedema. O pico da velocidade sistólica também foi significativamente maior com o uso de meia, comparado sem uso de meia, aos 30 e 60 minutos. A velocidade média do fluxo e fluxo total do volume foram maiores aos 60 e 120 minutos com o uso de meia.
Olsen et al. (2019)^22^	Não houve diferença significativa para TVP/TEP.	A diferença na circunferência do tornozelo foi 5 mm maior após o voo em comparação a antes do voo, IQR = 0–9, p = 0,001. As pernas vestindo MECG tiveram uma diminuição mediana na circunferência do tornozelo de 2 mm (p = 0,004) durante o voo, enquanto as pernas sem MECG tiveram aumento médio de 2 mm (p = 0,01). A diferença entre as circunferências da panturrilha antes e após o voo foi de 5 mm, IQR = 1–12, p < 0,001. Depois do voo, as pernas que usavam meia de compressão tiveram uma redução da circunferência da panturrilha de 3 mm (p = 0,007), enquanto a circunferência da panturrilha aumentou nas pernas sem MECG com mediana de 3 mm (p = 0,02).
		DOR E DESCONFORTO: Não houve mudança significativa na dor ou desconforto entre as pernas pós-voo em comparação a antes do voo. Embora a escala analógica visual da dor não tenha aumento durante o voo, houve um aumento na escala visual analógica para desconforto de 1 mm nas pernas com MECG (p = 0,005).

MECG = meias elásticas de compressão graduada; TVP = trombose venosa profunda; TEV = tromboembolismo venoso; TEP = tromboembolismo pulmonar; IQR = amplitude interquartil.

**Tabela 3:** Avaliação da qualidade metodológica dos ensaios clínicos randomizados através do instrumento *Consolidated Standards of Reporting Trials* (CONSORT).

Autor (ano)	Itens não cumpridos no CONSORT
Scurr et al. (2001)^17^	Item 23: não informa número de inscrição e nome do estudo clínico registrado;
	Item 24: não informa onde o protocolo completo do estudo clínico pode ser acessado.
Belcaro et al. (2002)^18^	Item 8a: não descreve o método utilizado para a geração de sequência randomizada de alocação;
	Item 8b: não descreve o tipo de randomização e detalhes de qualquer restrição;
	Item 11a e 11b: não descreve se houve cegamento após as intervenções serem atribuídas e como;
	Item 15: não há tabela apresentando os dados de base demográficos e características clínicas de cada grupo;
	Item 17a: não mostra o intervalo de confiança;
	Item 20: não demonstra limitações do estudo clínico, abordando fontes de potenciais vieses, imprecisão e, se pertinente, relevância das análises.
Belcaro et al. (2003)^14^	Item 1 a: não identifica no título como um estudo clínico randomizado;
	Item 8b: não detalha o tipo de randomização;
	Item 9: não detalha o mecanismo utilizado para implementar a sequência de alocação randomizada;
	Item 10: não informa quem gerou a sequência de alocação randomizada, quem inscreveu os participantes e quem atribuiu as intervenções aos participantes;
	Item 11a e 11b: não detalha se foi realizado cegamento e quem foi cegado após as intervenções serem atribuídas;
	Item 17a: não especifica o intervalo de confiança, apenas o valor de p;
	Item 24: não especifica onde o protocolo completo do estudo clínico pode ser acessado.
Cesarone et al. (2003)^19^	Item 1a: não identifica no título como um estudo clínico randomizado;
	Item 3a: não descreve o estudo clínico (paralelo, fatorial);
	Item 8b: não demonstra o tipo de randomização;
	Item 9: não especifica o mecanismo utilizado para implementar a sequência de alocação randomizada;
	Item 10: não especifica quem gerou a sequência de alocação randomizada, quem inscreveu os participantes e quem atribuiu as intervenções aos participantes;
	Item 11a e 11b: não especifica se houve cegamento, após as intervenções serem atribuídas;
	Item 17a: não especifica o intervalo de confiança, apenas o valor de p;
	Item 17b: não especifica o tamanho dos efeitos absolutos e relativos, apenas o valor de p;
	Item 24: não especifica onde o protocolo completo do estudo clínico pode ser acessado;
Cesarone et al. (2003)^20^	Item 3a: não descreve o estudo clínico (paralelo, fatorial);
	Item 8b: não descreve o tipo de randomização e detalhes de qualquer restrição;
	Item 9: não especifica o mecanismo utilizado para implementar a sequência de alocação randomizada, descrevendo os passos seguidos para a ocultação da sequência;
	Item 10: não detalha quem gerou a sequência de alocação randomizada, quem inscreveu os participantes e quem atribuiu as intervenções aos participantes.
Hagan et al. (2008)^21^	Item 9: não descreve o mecanismo utilizado para implementar a sequência de alocação randomizada, descrevendo os passos seguidos para ocultação da sequência até as intervenções serem atribuídas;
	Item 11a e 11b: não se aplica ao estudo;
	Item 12b: não mostra os métodos para análises adicionais, como análises de subgrupo e análises ajustadas;
	Item 14b: não cita os motivos para o estudo ter sido finalizado ou interrompido;
	Item 17b: não se aplica ao estudo em questão.
Charles et al. (2011)^13^	Item 3a: não descreve o estudo clínico;
	Item 11a e 11b: não especifica o cegamento;
	Item 14a: não define datas de recrutamento e períodos de acompanhamento;
	Item 23: não identifica número de inscrição e nome do estudo clínico registrado;
	Item 24: não identifica onde o protocolo completo do estudo clínico pode ser acessado.
Olsen et al. (2019)^22^	Cumpriu todos os itens propostos de acordo com a classificação CONSORT.

**Tabela 4:** Qualidade da evidência do desfecho avaliado.

Desfecho	N° de participantes dos estudos	Qualidade da evidência (GRADE)
Edema	1.311	Alta
Incidência de TVP/TEP	2.022	Moderada

GRADE = *Grading of Recommendations Assessment, Development and Evaluation*; TVP = trombose venosa profunda; TEP = tromboembolismo pulmonar.

## DISCUSSÃO

Esta revisão tem o objetivo de verificar a existência de evidências que comprovem os benefícios do uso de MECG em viagens aéreas de longa duração na redução da incidência de TEV e de edema de MMII. Apesar da incidência de TVP na população geral ser baixa, ela vem aumentando significativamente e torna-se ainda maior em pacientes considerados de alto risco, variando de 4 a 10%, sendo, portanto, aconselhada sua profilaxia^17^.

As MECG são consideradas um método de prevenção de TEV com menos efeitos colaterais quando comparadas a farmacoprofilaxia^13^^,^ 18. De acordo com alguns estudos, existe um efeito estatisticamente significativo naqueles que usam MECG comparados aos que não usam, reduzindo o risco de TVP em aproximadamente 90%^23^.

O sinal mais comum da TVP de MMII é o edema, observado em indivíduos que viajam por mais de 2 a 4 horas, por conta da restrição de movimento e imobilidade. Esse sinal pode ser exacerbado em pacientes portadores de doenças, como doença venosa crônica, microangiopatia diabética e insuficiência cardíaca, e é muitas vezes negligenciado, chegando a ser considerados normais pelos próprios passageiros após ficarem sentados por algumas horas^17^^-^ 20.

Por conta disso, medidas profiláticas no intuito de mitigar o risco de TEV têm sido estudadas, buscando reduzir a ocorrência de efeitos adversos. A presente revisão sistemática identificou estudos que relatam uma redução de incidência de TEV com o uso de MECG comparado ao não uso, cuja qualidade da evidência pode ser considerada moderada. Essa medida não farmacológica está associada a melhora da hemodinâmica venosa do membro, determinadas por medidas do pico da velocidade sistólica, volume médio de fluxo e volume de fluxo total na veia poplítea. Os estudos revisados apontam aumento da velocidade do fluxo sanguíneo nos MMII em cerca de 0,35 cm/s^14^.

É importante ressaltar que a interpretação desse desfecho pode ter sido limitada pela impossibilidade de cegar o sonógrafo durante o exame de ultrassom da veia poplítea, após alocação dos participantes. Mesmo assim, os resultados obtidos sugerem aumento do fluxo sanguíneo venoso dos MMII com o uso das MECG, sendo essa estratégia útil na redução do risco de TEV tanto em pacientes saudáveis quanto naqueles com doença venosa crônica^14^^,^ 17. Estudos demonstraram que a formação de trombos pode ser amenizada durante o uso da MECG ao aumentar a pressão perivascular e reduzir o contato dos fatores teciduais, mesmo em indivíduos de baixo-médio risco para TVP^18^^,^ 21 ^,^ 24.

Alguns estudos também evidenciaram que o edema é muito comum e bastante observado em quase todos os sujeitos que realizam viagens aéreas de longa duração, mesmo naqueles saudáveis. Foi demonstrado que pequenos aumentos quantitativos na circunferência do tornozelo já podem ser associados a percepções de edema nos MMII e a queixas de dor e desconforto, podendo o uso de MECG impedir o seu desenvolvimento. Ademais, os estudos indicam que o edema tende a aumentar com a duração do voo^18^^,^ 19 ^,^ 24, porém pesquisas mais recentes já verificaram a presença desse desfecho em voos mais curtos, de até 3 horas de duração, podendo também aumentar o risco para TEV já que, muitas vezes, o edema pode sobrevir tal evento, sendo o seu sinal mais comum. Recentemente Olsen et al.^22^ observaram uma redução do edema de MMII com o uso de MECG em uma amostra de indivíduos jovens saudáveis. Nesse estudo, a idade foi considerada um fator de risco maior para a sua formação, podendo, consequentemente, haver maior benefício do uso de MECG^22^.

Os estudos não demonstraram diferença significativa na dor ou desconforto em voos de curta duração, comparando MMII com ou sem MECG. Encontrou-se apenas um discreto aumento no desconforto, medido pela escala analógica visual de dor, de 1 mm nas pernas com MECG, fato que pode não ser relevante. Esse desconforto pode estar relacionado com a idade dos participantes, pois eram jovens e podiam não estar acostumados com o uso de MECG, como também com a pressão exercida pela meia, podendo não ter relação direta com o edema^22^. Diante disso, sugere-se que estudos futuros devam investigar a associação da dor e desconforto relacionado ao edema de MMII em viagens de curta duração.

É válido comparar o estudo realizado em voos de menor duração (3 horas) com aqueles realizados em voos de maior duração (acima de 5 horas). Percebeu-se que, nos voos de menor duração, houve maior diferença na redução da circunferência do tornozelo após o uso das MECG e pouca diferença na melhora da dor e do desconforto^22^. Já nos voos de maior duração, observou-se uma menor diferença na redução da circunferência do tornozelo após o uso das MECG, mas uma diferença significativa na melhora da dor e do desconforto^21^. Além da duração do voo, que exerce influência direta nas medidas da circunferência do tornozelo, podem ter contribuído com esses achados a idade dos passageiros, visto que os mais velhos podem ser mais sensíveis à formação de edema ou menos sensíveis à compressão gerada pelas MECG^21^^,^ 22. Foi observado também que alguns estudos incluíram, independente do uso ou não das MECG, recomendações como realização de exercícios durante os voos, sugestões de dieta — como estímulo a beber água e evitar alimentos salgados — e redução da quantidade de bagagem a bordo para manter espaço livre entre as pernas^24^, o que pode também ter contribuído na redução de TVP e edema.

Observou-se algumas limitações dos estudos selecionados, como terem sido conduzidos em ambientes de pesquisa sob controle de condições e em participantes saudáveis, resultando em achados que podem não ser generalizáveis para participantes com doença venosa ou arterial ou hábitos corporais diferentes^21^. Assim, sugere-se que estudos futuros investiguem o efeito das MECG sobre o edema em MMII de passageiros com risco aumentado de sua formação (idade avançada, doenças venosas) e sobre a redução da incidência de TEV, em voos com duração inferior a 4 horas. Essa afirmativa é reforçada pelo fato de que os participantes do estudo de Olsen et al.^22^ eram jovens, o que pode limitar a validade externa dos resultados quando considerados passageiros idosos e com comorbidades. Como a idade é um fator de risco para edema em MMII^25^^,^ 26, os passageiros mais velhos podem ter a sua formação mais pronunciada e, consequentemente, obter maior benefício do uso de MECG, mesmo durante voos de curta duração^22^. Além disso, é válido salientar que a avaliação da ocorrência de TEV requer um número maior de sujeitos e observações mais prolongadas, o que talvez tenha dificultado o registro de sua incidência entre os estudos selecionados^18^.

## CONCLUSÃO

Esta revisão sistemática demonstra a existência de evidências científicas de alta qualidade para a prevenção de edema e de moderada qualidade para a redução da incidência de TEV com o uso de MECG durante viagens aéreas de longa duração. Foi observada a redução do edema após o uso de MECG em voos com duração de 3 horas, sugerindo que passageiros com edema nos MMII, sentindo dor ou desconforto, provavelmente se beneficiariam do seu uso. Entretanto, são necessários novos ensaios clínicos randomizados investigando o efeito do uso de MECG em voos de menores durações para verificar se há interferência na redução da incidência de TEV e sinais como edema, dor e desconforto.
